# Isolation of Shiga toxin-producing Escherichia coli from sheep faecal samples: bacteriological findings

**DOI:** 10.1099/acmi.0.001004.v3

**Published:** 2025-07-21

**Authors:** Xhelil Koleci, Pëllumb Zalla, Majlind Sulçe, Tristan Russell, Gerald Muça, Egon Andoni, Séamus Fanning

**Affiliations:** 1 Faculty of Veterinary Medicine, Agricultural University of Tirana, Tirana, Albania; 2 Centre for Experimental Pathogen Host Research (CEPHR), University College Dublin, Dublin D04 E1W1, Ireland; 3 UCD-Centre for Food Safety, School of Public Health, Physiotherapy and Sports Science, University College Dublin, Belfield, Dublin D04 N2E5, Ireland

**Keywords:** antimicrobial susceptibility, farm biosecurity, seasonality of shedding, sheep reservoirs, Shiga toxin-producing *Escherichia coli*

## Abstract

Shiga toxin-producing *Escherichia coli* (STEC) poses a significant public health risk due to its zoonotic potential and association with foodborne outbreaks. This study investigates the presence of STEC in faecal samples collected from sheep, focusing on bacteriological methods for isolation and preliminary characterization. Growth on selective agar showed that 90 of 140 faecal samples were positive for STEC. The total average prevalence was 64.3% (95% CI 56.1–71.7%). The highest prevalence was 71.4% (54.9–83.7%), recorded in summertime, while the lowest was 51.4% (35.6–67%) in the autumn. Selected isolates were found to be resistant to commonly used antibiotics, with isolates expressing a resistant phenotype to two, three or more of the tested antibiotics. Based on the Biocheck.UGent system for risk-based assessment of farm biosecurity, the total, internal and exterior average farm biosecurity scores were substantially lower than the world average.

## Data Summary

All relevant data used in this study are fully presented within the manuscript or supplementary information section, and no data upload to an external repository is required. The tool used for assessing farm biosecurity is available here: https://biocheckgent.com/en.

## Introduction

Shiga toxin-producing *Escherichia coli* (STEC) produces Shiga toxins (Stx1 and Stx2), leading to severe human illnesses such as haemorrhagic colitis and haemolytic uremic syndrome (HUS) [1, 2]. Sheep, goats, cattle and a range of wild animals are recognized as asymptomatic carriers of STEC, shedding these bacteria in their faeces [3,6]. An Italian investigation of human STEC infections causing HUS identified one patient of 43 who most likely contracted STEC during a holiday in Albania, but an in-depth investigation into this case was not conducted [7] . STEC is also a major contributor to foodborne illness, with signs ranging from minor intestinal discomfort and haemorrhagic diarrhoea to more serious illnesses, including HUS, end-stage renal failure and in some cases death [1, 5,8, 9]. According to WHO’s assessment on the worldwide burden of foodborne illness, STEC was responsible for over 1.2 million illnesses, 128 fatalities and around 13,000 disability-adjusted life years in 2010 [10]. In humans, STEC infection was the third most frequently reported foodborne gastrointestinal illness in the European Union (EU). In 2023, there were 10,217 confirmed cases of human STEC infection, resulting in an EU notification rate of 3.1 cases per 100,000 people. This was a 29.2% increase from 2022 (2.4 cases per 100,000 population), but the number of foodborne cases has decreased from 408 in 2022 to 270 in 2023 [1]. The overall trend for STEC infections in EU member states showed an increase between 2019 and 2023 [1].

In 2023, STEC was isolated from 354 food sampling units out of 16,117 using the ‘objective sampling’ strategy, meaning samples were randomly selected at a sufficient size to be representative of the overall population, then tested with methodologies for detecting any STEC [1]. The ‘ready-to-eat’ food category included 6,863 sampling units reported by 19 member states, of which 1.0% were positive [1]. Most positive sampling units were from the ‘milk and milk products’ (2.0% positive) and ‘meat and meat products’ (1.3% positive), but the highest food category with the greatest proportion of positives was ‘bakery items’, where 3 (6.7%) of the 45 sampling units tested positive for STEC [1]. Out of the 9,254 ‘non-ready-to-eat’ samples reported by 21 EU states, 3.1% tested positive, with ‘meat and meat products’ having a positivity of 3.8% [1], which is less than the 4.8% STEC positivity for ground meat collected from Tirana market in 2010/2011 [11] or the 28.57% positivity for chicken meat from Tirana in 2015 [12]. STEC transmission to humans can occur *via* contaminated food, direct contact with animals or environmental contamination. Cattle, sheep and goats are the known primary reservoirs of STEC; however, wild species can also serve as reservoirs and play a role in infection transmission within ecosystems [4]. The high prevalence of STEC in Albanian meat products compared to other EU states could be an indication of high overall prevalence in STEC reservoirs and highlights a potential public health concern via zoonotic transmission or from contaminated foods, though the below average consumption of meat in Albania could confound this [13].

To the author’s knowledge, there are no published studies into STEC prevalence in Albanian livestock, so a comparison with EU states cannot be carried out. This study determined whether sheep sampled in Albania from December 2023 to November 2024 harbour STEC by isolating and identifying STEC from healthy sheep faeces. Antibiotic resistance profiles were then determined for randomly selected STEC isolates. In addition, the shedding seasonality dynamic and primary sheep farm biosecurity level were investigated to associate these with changes in STEC prevalence.

## Methods

### Farm selection and sampling rationale

Seven medium-sized sheep farms were selected from six different districts in Albania: Korça, Vlora, Kukës, Dibra, Shkodra and Mamurras. These districts were chosen because they collectively account for over 54% of the national sheep population [14], thus ensuring that the study covers the most representative regions in terms of sheep density and management practices. Notably, two farms were included from the Korça district, based on the recommendation of the local veterinary expert and the region’s high concentration of sheep farms.

From each of the seven farms, five sheep were randomly selected for sampling. This number was chosen as a practical balance between logistical feasibility and the need to obtain representative data from each herd. Sampling was conducted on four separate occasions over the course of a year – once per season (spring, summer, autumn and winter) – between December 2023 and November 2024. This seasonal sampling approach was designed to capture potential temporal variations in health status, disease dynamics and environmental influences.

### Sample collection

A total of 140 faecal samples were taken from sheep’s rectums using sterile gloves. Each sample was carefully placed in a sterile container to maintain its integrity and avoid contamination. Following collection, the samples were promptly stored in a cool box to preserve their biological viability before being transported to the laboratory for additional microbiological and molecular analysis at controlled temperatures.

### Bacteriological isolation

Samples were processed within 24 h of collection. Briefly, ~3–5 g of faecal material collected from each animal was homogenized, and 1 g was suspended in 5 ml of physiological solution before being transferred into 35 ml of liquid broth supplemented with novobiocin and incubated at 41.5 °C for 5 h, followed by incubation at 37 °C for 18–24 h (overnight). Briefly, ~10 µl of enriched samples were then streaked onto CHROMagar^™^ STEC (CHROMagar^™^ STEC, www.CHROMagar.com), a chromogenic medium used to isolate and differentiate *E. coli* O157 in clinical and food samples. Interpretation of results was based on typical colony appearance on CHROMagar^™^ STEC medium. Colonies were classified as strong, medium or weak based on the degree of mauve colour observed, which ranged from dark to medium mauve, lighter mauve to pinkish and very pale pink (Table 1).

**Table 1.** Criteria used to classify the presence of *E. coli* O157 colonies and other bacterial growth

Micro-organism	Typical colony appearance
*E. coli* O157	**Mauve**
Coliforms	Metallic blue
*Proteus*	Colourless to grey

### Antimicrobial susceptibility testing

STEC isolates were tested for their susceptibility to a panel of ten antibiotics commonly used in clinical and veterinary practice representing different classes using the Kirby-Bauer disc diffusion method, following published guidelines [15] (EUCAST https://www.eucast.org/). Four antibiotic classes were used: (a) aminoglycosides (amikacin), (b) beta-lactams (amoxicillin, cefazolin, cefixime and cefoxitin), (c) fluoroquinolones (ciprofloxacin, enrofloxacin and nalidixic acid) and (d) tetracyclines (doxycycline and tetracycline). Antibiotics [with quantities (µg) in parenthesis] included amikacin AK (30), amoxicillin AX (25), cefazolin CZ (30), cefixime CFM (5), cefoxitin FOX (30), ciprofloxacin CIP (5), doxycycline DO (30), enrofloxacin ENR (30), nalidixic acid NA (30) and tetracycline TE (30). Diagram S1 (available in the online Supplementary Material) illustrates the methods employed for isolating STEC from faecal samples. Criteria used to determine susceptibility are shown in Table 2.

**Table 2.** Criteria (mm) used to determine susceptibility and resistance of the isolates. The top two rows define susceptibility and resistance breakpoints for each antibiotic

Threshold	Amicacin	Amoxicillin	Cefazolin	Cefixime	Cefoxitin	Ciprofloxacin	Doxycycline	Enrofloxacin	Nalidixic acid	Tetracycline
Susceptible	18	19	17	19	22	18	25	19	21	19
Resistant	15	13	14	16	19	15	22	16	16	15

### Biosecurity assessment

The level of biosecurity on sheep farms was assessed using the BioCheck.UGent Small Ruminant Module, an online standardized risk-based scoring tool developed by Ghent University (https://biocheckgent.com/en) [16,17]. This tool evaluates biosecurity practices through a structured questionnaire covering internal and external biosecurity measures. Scores are calculated based on responses and benchmarked against international reference data, allowing for objective comparison and identification of critical weaknesses.

## Results

### Detection of *E. coli* O157

**Fig. 1.**
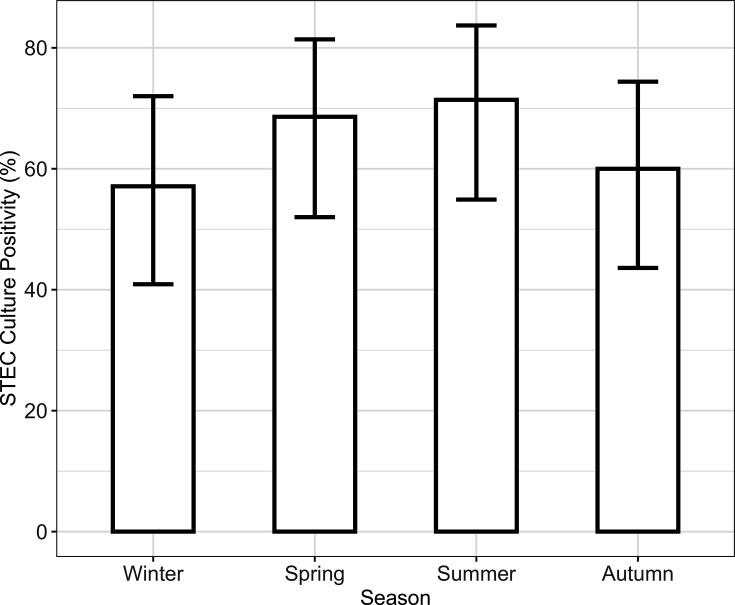
STEC positivity according to season. Error bars represent the 95% CI. STEC culture positivity varies seasonally, increasing in the spring and summer and declining in the winter and autumn, with some level of uncertainty illustrated by the error bars.

All faecal samples produced mixed bacterial colonies, and 90 of the 140 collected samples were phenotypically STEC positive, generating a prevalence of 64.3% (95% CI 56.1–71.7%). The highest positivity rate of 25 (71.4%) was recorded during the summer, suggesting an increase in *E. coli* O157 shedding during warm weather. Positivity was lowest in winter (57.1%) and somewhat higher in autumn (60.0%), indicating seasonal patterns in bacterial shedding. However, the overlap of CI between seasons shows that the difference in positivity was not statistically significant (Table S1, Fig. 1).

**Fig. 2.**
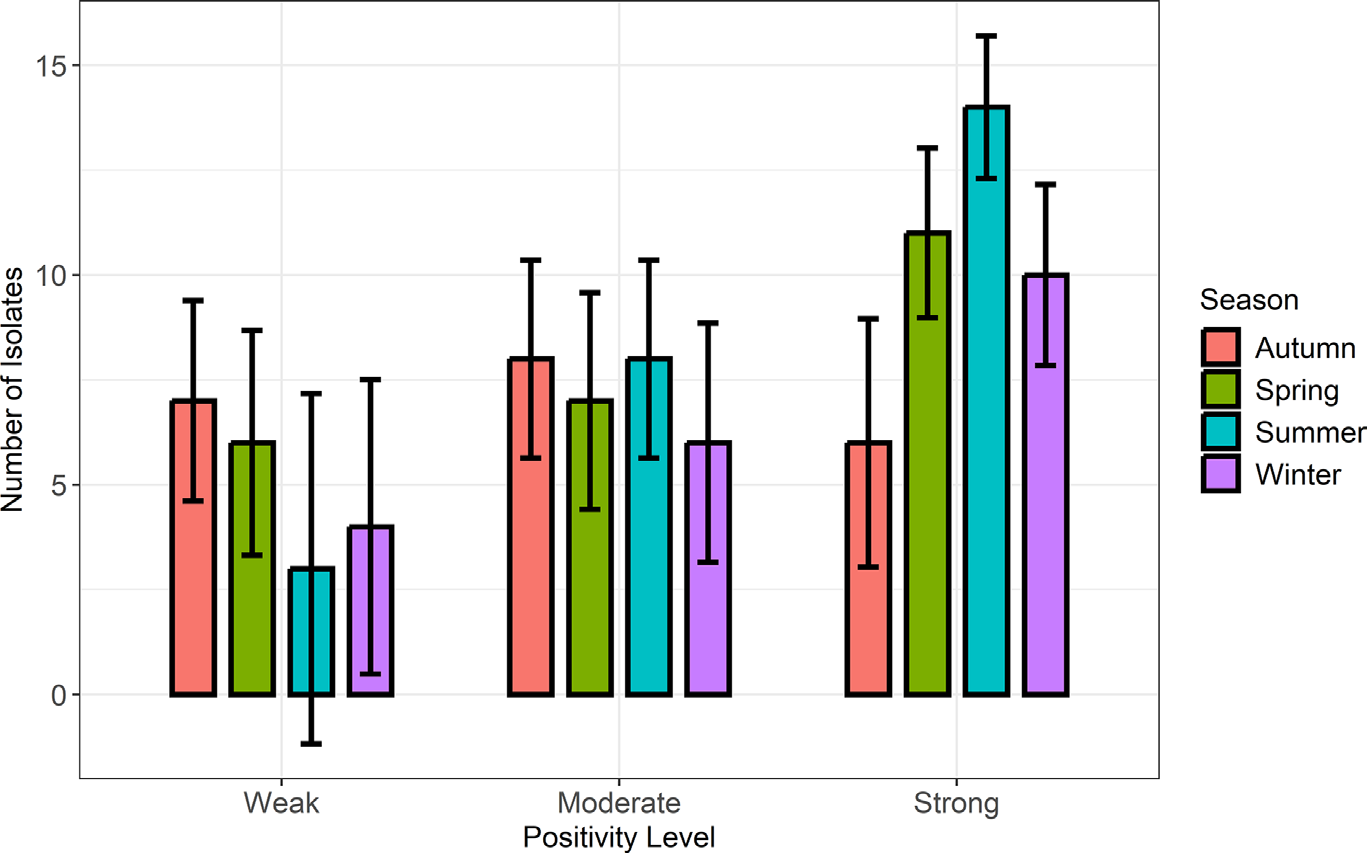
Isolation of *E. coli* O157 and its positivity level across seasons. This bar chart illustrates the seasonal distribution of *E. coli* O157 isolates at various positivity levels (weak, moderate and strong). The bars are colour-coded according to season (winter, spring, summer and autumn), and the *y*-axis shows the number of isolates.

Summer had the highest number of strong positive samples (14 animals), whereas the fall season had the lowest number (6 animals). Moderately positive samples were continuously high in the spring (7), summer (8) and autumn (8), indicating sustained detection rates over these seasons. Weakly positive samples were highest in the autumn (7) and lowest in the summer (3), demonstrating the seasonal variation in bacterial burden. The CI shows variability in positivity rates, and their overlap between seasons shows that although a trend may exist, it is not statistically significant (Fig. 2).

### Antibiotic susceptibility testing

Antibiotic susceptibility tests were performed for a panel of 10 antibiotics using 16 randomly selected suspected STEC colonies (Table S2). Resistance was determined for each isolate using the Kirby-Bauer disc diffusion assay (Table S3). Resistance to several antibiotics was high, especially amoxicillin (87.5%), cefixime (87.5%), enrofloxacin (87.5%), nalidixic acid (81.3%) and ciprofloxacin (81.3%). In total, 75% of isolates were resistant to doxycycline and 68.8% to tetracycline (Fig. S1). Antibiotics such as cefoxitin (56.3%), cefazolin (62.5%) and amikacin (68.8%) had lower resistance rates than others, but susceptibility to these antibiotics was limited (Fig. S1). Cefoxitin, with a susceptibility rate of 43.8%, stands out as having relatively higher efficacy than others, followed by cefazolin, amikacin and tetracycline, which each had 31.3% susceptibility (Table 3).

**Table 3.** Resistance of STEC isolates to screened antibiotics

Antibiotic	**Percentage (No.**)	
	**Susceptible**	**Resistant**
Amikacin	31.2 (5)	68.8 (11)
Amoxicillin	12.5 (2)	87.5 (14)
Cefazolin	37.5 (6)	62.5 (10)
Cefixime	12.5 (2)	87.5 (14)
Cefoxitin	43.7 (7)	56.3 (9)
Ciprofloxacin	18.7 (3)	81.3 (13)
Doxycycline	25.0 (4)	75.0 (12)
Enrofloxacin	12.5 (2)	87.5 (14)
Nalidixic acid	18.7 (3)	81.3 (13)
Tetracycline	31.2 (5)	68.8 (11)

**Fig. 3.**
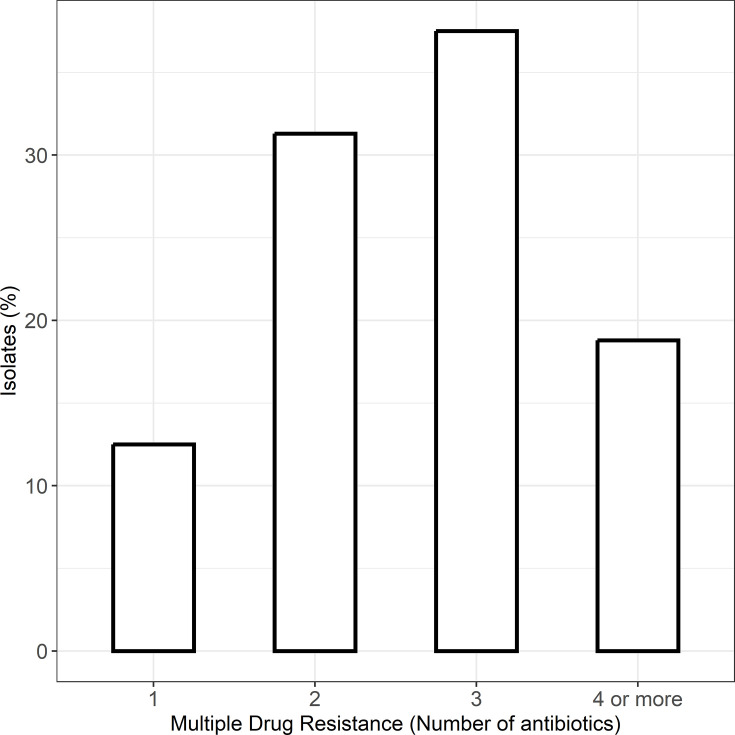
Proportion of MDR STEC isolates.

Several STEC were classified as expressing a multiple drug resistant (MDR) phenotype, with 31.3% resistant to two antibiotics, 37.5% resistant to three antibiotics and 18.3% resistant to more than three types of tested antibiotics (Table S4, Fig. 3). Only 12.5% of isolates were resistant to a single antibiotic, showing most isolates developed multiple resistance.

### Farm biosecurity

**Fig. 4.**
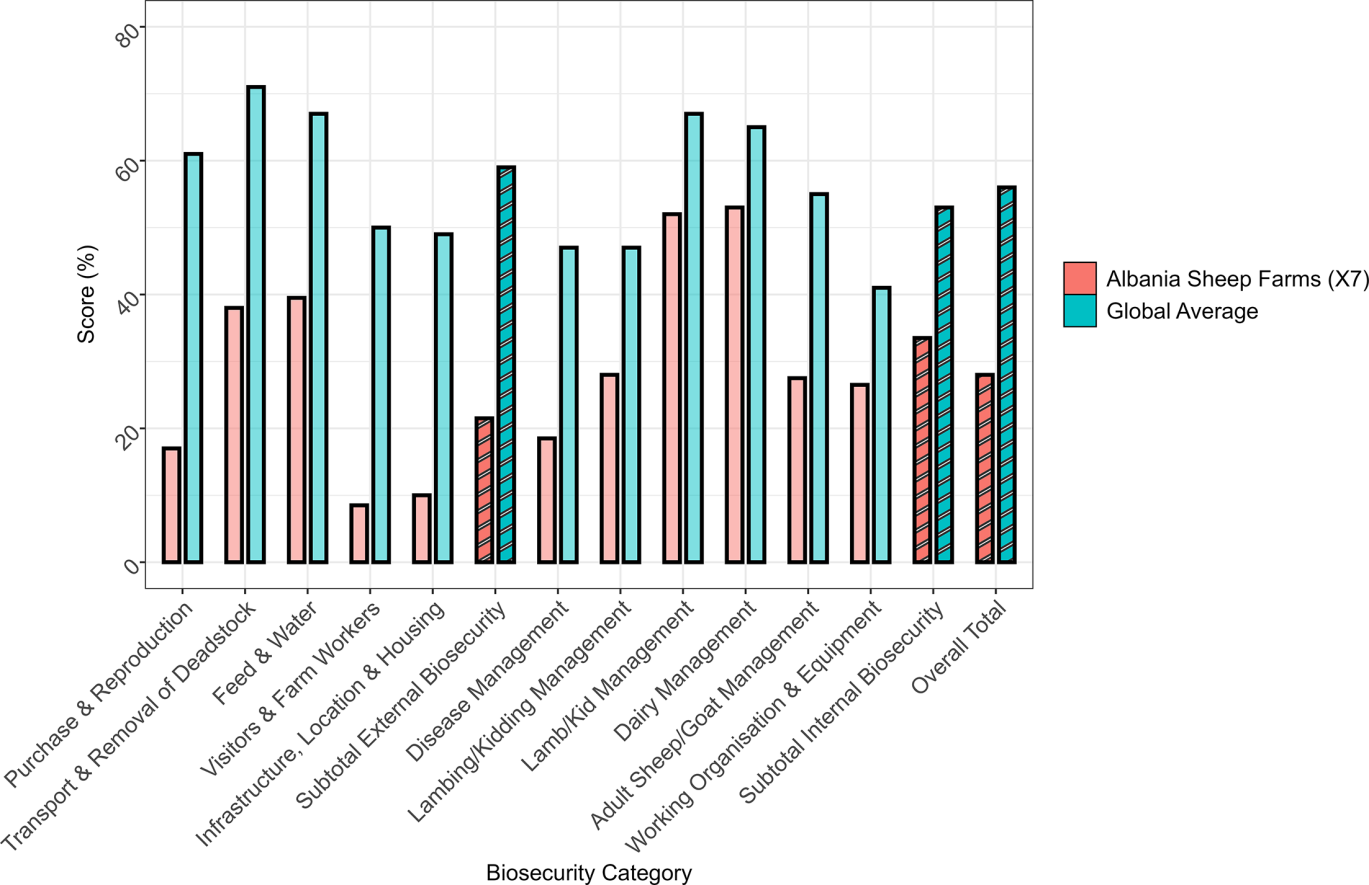
Internal, external and total biosecurity scores (%) are based on the Biocheck.UGent system [16]. Bars representing subcategories are opaque than those representing internal, external and overall totals, which are striped.

Biosecurity is vital for protecting both animal and public health by preventing the introduction and spread of infectious diseases, including zoonoses. One of the most concerning findings from the BioCheck.UGent assessment for external biosecurity was the very low score of 17% in the ‘Purchase and Reproduction’ category, indicating major gaps in protocols for acquiring new animals and managing reproductive health, which are key risk points for disease entry (Fig. 4). Weak carcass disposal practices further increase the risk of spreading zoonotic pathogens between and within farms. Feed and water hygiene scored just 39.5%, well below the international benchmark of 67% (Fig. 4). Poor quality control in this area can lead to environmental contamination and greater exposure of animals to pathogens transmissible to humans, such as STEC. STEC can persist in contaminated water and feed, and infected animals often remain asymptomatic, posing a hidden risk to handlers and consumers. Alarmingly, access control to farms scored only 8.5% (vs. 50% globally), allowing greater risk of disease introduction *via* people, vehicles or equipment (Fig. 4). This is particularly hazardous with pathogens like STEC, where even limited contamination can lead to outbreaks affecting farm workers, surrounding communities or the food chain. A score of just 10% in ‘Infrastructure, Location and Housing’ (vs. 49% globally) highlights critical weaknesses in farm layout, drainage and housing – all factors that support pathogen persistence in the environment (Fig. 4).

The average internal biosecurity score across sheep farms was 21.5%, far below the global average of 59%, indicating systemic on-farm disease management failures (Tables S5 and S6, Fig. 4). The ‘Disease Management’ score of 18.5% (vs. 47% globally) reflects poor surveillance, underreporting and limited containment measures (Fig. 4). Lambing practices, a high-risk period for transmission, also scored poorly (28 vs. 47% globally) (Fig. 4). Of all external and internal biosecurity categories, only ‘Lamb/Kid Management’ (52 vs. 57% globally) and ‘Dairy Management’ (53 vs. 65% globally) came close to global standards, though improvement is still needed (Fig. 4). In contrast, ‘Adult Sheep/Goat Management’ and ‘Working Organization and Equipment’ scored just 27.5 and 26.5%, respectively, pointing to serious lapses in animal health control and equipment hygiene – both crucial in limiting zoonotic threats such as STEC, *Brucella* sp., *Listeria* sp. and *Coxiella burnetii* (Fig. 4). These findings reveal major gaps in biosecurity, representing not only a threat to livestock productivity but also a significant public health concern due to increased risk of zoonotic disease emergence and spread.

## Discussion

STEC is a prominent zoonotic disease that has serious consequences for veterinary public health. Understanding its prevalence and molecular features in animal herds is critical for disease prevention and management. In Albania, data on STEC occurrence in livestock are sparse, and this study is the first to investigate its existence in sheep, which can operate as asymptomatic reservoirs of the bacteria. This study attempts to close a key knowledge gap in STEC epidemiology by analysing sheep faeces. The findings will provide useful insights for targeted initiatives to reduce transmission risks and improve food safety in Albania.

To identify possible STEC-positive sheep, CHROMagar O157 was used, as it selectively supports the growth of *STEC*. Sorbitol MacConkey (SMAC) agar is another option for *E. coli* O157:H7 culture [18,20] but CHROMagar O157 significantly improves routine detection of *E. coli* O157, exceeding SMAC in terms of sensitivity, efficiency and cost-effectiveness [21]. The high prevalence of STEC in sheep, 64.3% (95% CI 56.1–71.7%), recorded in this study agrees with previous studies assessing animal reservoirs and transmission routes [5,8, 22,24, 25]. Ruminants, notably cattle, have been identified as the predominant reservoir of *E. coli* O157:H7, with sheep and goats also contributing to its maintenance and spread. The bacterium remains asymptomatic in these food-producing hosts, making diagnosis and control difficult. Human infections are primarily caused by consumption of contaminated foods such as undercooked ground beef, unpasteurized dairy products or contaminated fresh fruit [1,26]. Less common methods of human infection include direct contact with animals or their faeces, contaminated water sources and person-to-person dissemination. The results confirm the presence of STEC in sheep faeces, reinforcing their role as potential reservoirs.

Results assessing the seasonal shedding dynamics of STEC should be interpreted with caution because CI overlapped between seasons. However, there was evidence of increased shedding throughout the summer, which could be attributed to environmental conditions such as temperature and humidity. It has previously been shown that these conditions promote *E. coli* O157 survival and transmission [27]. The lower incidence in winter and autumn may indicate diminished environmental shedding or bacterial activity during the colder months. The high-shedding rates in the summer coincide with peak agricultural and recreational activity, raising the risks of human exposure to *E. coli* O157 from animals. These findings highlight the importance of focused biosecurity measures during high-risk seasons, notably summer, to prevent contamination of feed, water and the environment and increased monitoring frequency to effectively detect and manage *E. coli* O157 shedding hotspots [1,8].

Improved farm-level cleanliness procedures during high-shedding periods should be implemented to limit the spread of bacteria [28]. Interventions to reduce zoonotic risk include food changes to lower *E. coli* O157 burdens in animals; education of farmers and the public about the heightened risk of *E. coli* transmission during the summer months; focus on cleanliness and animal handling safety. Studies in other European countries support the findings reported here in showing strong seasonal fluctuations in *E. coli* O157 shedding, with summer posing the biggest danger. This emphasizes the significance of tailored management techniques and increased attention during warmer months to prevent the public health hazards linked with zoonosis [8].

The results indicate that the tested isolates expressed an MDR phenotype, limiting treatment options. Amikacin and cefoxitin may still have some therapeutic benefit, but they should be administered with caution, preferably based on susceptibility testing. The high resistance rates to routinely used antibiotics (such as amoxicillin, ciprofloxacin and cefixime) indicate the need for rigorous management of antibiotic prescribing practices. Antimicrobial resistance patterns observed in this study highlight the need for prudent antibiotic use in livestock management to mitigate public health risks [26,29]. Options to overcome MDR STEC include restricting the use of antibiotics that are extremely resistant in clinical and veterinary settings, identifying combination therapies or novel antibiotics to treat resistant pathogens and continuously monitoring antimicrobial resistance trends for informed decision-making. Improved biosecurity can also reduce the incidence of MDR [30].

This study highlighted the high proportion of isolates that appeared resistant to several antibiotics. Antibiotics belonging to the penicillin (amoxicillin and cefixime) and (fluoro)quinolone (enrofloxacin and nalidixic acid) groups show many resistant cases, while cefoxitin and cefazolin may remain effective. Some STEC isolates were susceptible to amikacin, tetracycline and doxycycline. Four samples did not express an MDR phenotype, while 68.75% were resistant to two or more tested antibiotics. This research highlights the importance of taking a proactive approach to managing and mitigating the threat posed by resistant bacterial species. Addressing these concerns is crucial for ensuring human and animal health [26,29].

Biosecurity scores of the seven selected sheep farms are much lower than the global averages in both external and internal biosecurity categories. This indicates significant gaps in farm management that may contribute to the spread of STEC and other veterinary and zoonotic infectious agents. The aggregate total biosecurity score (28 vs. 56% globally) indicates a systematic deficit in biosecurity efforts across farms. Internal biosecurity (33.5%) is slightly higher than external (28%), but both require improvement. Improved farm-level biosecurity, increased abattoir hygiene and consumer education on safe food handling and preparation techniques are among the strategies that should be employed. The authors emphasize the importance of ongoing research into *E. coli* O157:H7 ecology, host–pathogen interactions and novel intervention approaches to reduce its public health impact. Poor biosecurity raises the risk of zoonotic disease transmission, including STEC, and can increase disease prevalence, production losses and treatment expenses [31]. Addressing crucial areas such as visitor control, feed quality and disease management will help farms match global standards and reduce outbreak risk [1,2, 16].

This study highlights the role of sheep as STEC reservoirs in Albania, which could have public health implications associated with zoonotic transmission. Seasonal shedding dynamics, antibiotic resistance profiles and biosecurity gaps on farms indicate the need for improved monitoring and control approaches on sheep farms.

## Study limitations and next research work

The study primarily uses bacteriological approaches to identify STEC. Molecular techniques, such as PCR for specific virulence genes (stx1, stx2 and eae), may improve strain characterization. Due to the lack of historical data, the extent to which this cross-sectional study is representative of Albania STEC prevalence in general cannot be determined, but it will act as a benchmark. Although seasonal differences in STEC shedding were discovered, external environmental factors (such as humidity, feed changes and management practices) were not thoroughly investigated, which could influence the interpretation of seasonal trends. The study examined only a small number of antibiotics. A greater range of antimicrobial drugs, particularly those identified as critical for human medicine, may reveal a more detailed resistance profile. There was no epidemiological data linking detected STEC strains to human cases, making it difficult to assess the isolates' public health impact. To address the abovementioned constraints, further work is continuing, including testing the STEC isolates for virulence genes, sequencing and broadening the range of antimicrobial resistance panels, including detection of resistance genes.

## Supplementary material

10.1099/acmi.0.001004.v3 Uncited Supplementary Material 1.

## Abbreviations

EU: European Union
HUS: haemolytic uremic syndrome
MDR: multiple drug resistant
SMAC: Sorbitol MacConkey
STEC: Shiga toxin-producing *Escherichia coli*

## References

[1] European Food Safety Authority (EFSA), European Centre for Disease Prevention and Control (ECDC) (2024). The European Union One Health 2023 Zoonoses report. *EFS2*.

[2] Food and Agriculture Organization of the United Nations World Health Organization Rome (2022). Control measures for Shiga toxin-producing Escherichia coli (STEC) associated with meat and dairy products. https://openknowledge.fao.org/server/api/core/bitstreams/cad%209c000-24af-4f02-90e1-3a70ad6c5c9b/content.

[3] Asakura H, Makino S, Shirahata T, Tsukamoto T, Kurazono H (1998). Detection and genetical characterization of Shiga toxin-producing *Escherichia coli* from wild deer. Microbiol Immunol.

[4] Espinosa L, Gray A, Duffy G, Fanning S, McMahon BJ (2018). A scoping review on the prevalence of Shiga-toxigenic *Escherichia coli* in wild animal species. Zoonoses Public Health.

[5] McCarthy SC, Macori G, Duggan G, Burgess CM, Fanning S (2021). Prevalence and whole-genome sequence-based analysis of Shiga toxin-producing *Escherichia coli* isolates from the recto-anal junction of slaughter-age irish sheep. Appl Environ Microbiol.

[6] Ørskov F, Ørskov I, Villar JA (1987). Cattle as reservoir of verotoxin-producing *Escherichia coli* 0157:H7. Lancet.

[7] Ria T, Mancuso MC, Daprai L, Liporace MF, Gazzola A (2024). Vacation in Egypt associated with Shiga toxin-producing *Escherichia coli* infection in children and adolescents, northern Italy, 2023. Euro Surveill.

[8] Oporto B, Ocejo M, Alkorta M, Marimón JM, Montes M (2019). Zoonotic approach to Shiga toxin-producing *Escherichia coli*: integrated analysis of virulence and antimicrobial resistance in ruminants and humans. Epidemiol Infect.

[9] Parsons BD, Zelyas N, Berenger BM, Chui L (2016). Detection, characterization, and typing of Shiga toxin-producing *Escherichia coli*. Front Microbiol.

[10] WHO (2015). Foodborne Diseases Estimates. https://www.who.int/data/gho/data/themes/who-estimates-of-the-global-burden-of-foodborne-diseases.

[11] Gjurgji A, Sulaj K (2013). Occurrence of *E. coli* o:157 h:7 of ground meat samples collected from butcher shops in Tirana market. Albanian J Agric Sci.

[12] Daҫi A, Shehdula D, Ozuni L (2016). *E.coli.* in chicken meat in slaughterhouses and at retail shops in tirana. J Multidiscip Eng Sci Technol.

[13] Dosti D, Sinani O, Tila I (2021). Nutritional and Health Aspects of Food in the Balkans.

[14] INSTAT (2024). Albania. https://www.instat.gov.al/al/temat/bujq%C3%ABsia-dhe-peshkimi/blegtoria/publikimet/2024/statistikat-e-blegtoris%C3%AB-2023/.

[15] Markey B, Leonard F, Archambault M, Cullinane A, Maguire D (2013). Clinical Veterinary Microbiology.

[16] Gelaude P, Schlepers M, Verlinden M, Laanen M, Dewulf J (2014). Biocheck.UGent: a quantitative tool to measure biosecurity at broiler farms and the relationship with technical performances and antimicrobial use. Poult Sci.

[17] Biocheck.UGent Biocheck.UGent Home. https://biocheckgent.com/en.

[18] Bettelheim KA (1998). Reliability of CHROMagar O157 for the detection of enterohaemorrhagic *Escherichia coli* (EHEC) O157 but not EHEC belonging to other serogroups. J Appl Microbiol.

[19] Cooley MB, Jay-Russell M, Atwill ER, Carychao D, Nguyen K (2013). Development of a robust method for isolation of shiga toxin-positive *Escherichia coli* (STEC) from fecal, plant, soil and water samples from a leafy greens production region in California. PLoS One.

[20] March SB, Ratnam S (1986). Sorbitol-MacConkey medium for detection of *Escherichia coli* O157:H7 associated with hemorrhagic colitis. J Clin Microbiol.

[21] Church DL, Emshey D, Semeniuk H, Lloyd T, Pitout JD (2007). Evaluation of BBL CHROMagar O157 versus sorbitol-MacConkey medium for routine detection of *Escherichia coli* O157 in a centralized regional clinical microbiology laboratory. J Clin Microbiol.

[22] Al-Ajmi D, Rahman S, Banu S (2020). Occurrence, virulence genes, and antimicrobial profiles of *Escherichia coli* O157 isolated from ruminants slaughtered in Al Ain, United Arab Emirates. BMC Microbiol.

[23] Shahzad A, Ullah F, Irshad H, Ahmed S, Shakeela Q (2021). Molecular detection of Shiga toxin-producing *Escherichia coli* (STEC) O157 in sheep, goats, cows and buffaloes. Mol Biol Rep.

[24] Gencay YE (2014). Sheep as an important source of *E. coli* O157/O157:H7 in Turkey. Vet Microbiol.

[25] Ferens WA, Hovde CJ (2011). *Escherichia coli* O157:H7: animal reservoir and sources of human infection. Foodborne Pathog Dis.

[26] Ramos S, Silva V, Dapkevicius M de LE, Caniça M, Tejedor-Junco MT (2020). *Escherichia coli* as commensal and pathogenic bacteria among food-producing animals: health implications of extended spectrum β-lactamase (ESBL) production. Animals.

[27] Williams KJ, Ward MP, Dhungyel OP, Hall EJS (2015). Risk factors for *Escherichia coli* O157 shedding and super-shedding by dairy heifers at pasture. Epidemiol Infect.

[28] Ward C, Finical W, Smith K, Rounds JM, Klumb CA (2025). Ruminant-dense environments increase risk of reported Shiga toxin-producing *Escherichia coli* infections independently of ruminant contact. Appl Environ Microbiol.

[29] Ribeiro LF, Nespolo NM, Rossi GAM, Fairbrother JM (2024). Exploring Extended-Spectrum Beta-Lactamase (ESBL)-producing *Escherichia coli* in Food-producing animals and animal-derived foods. Pathogens.

[30] Mencía-Ares O, Argüello H, Puente H, Gómez-García M, Manzanilla EG (2021). Antimicrobial resistance in commensal *Escherichia coli* and *Enterococcus spp*. is influenced by production system, antimicrobial use, and biosecurity measures on Spanish pig farms. Porcine Health Manag.

[31] Layton DS, Choudhary A, Bean AGD (2017). Breaking the chain of zoonoses through biosecurity in livestock. Vaccine.

