# Supplementary material for: Isolation of Shiga toxin-producing Escherichia coli from sheep faecal samples: bacteriological findings
Source: Access Microbiol. 2025 Jul 21;7(7):001004.v3. doi: 10.1099/acmi.0.001004.v3 (PMC12284411; doi:10.1099/acmi.0.001004.v3)
Supplement: Uncited Supplementary Material 1. [file acmi-7-01004-s001.pdf]

Supplementary material

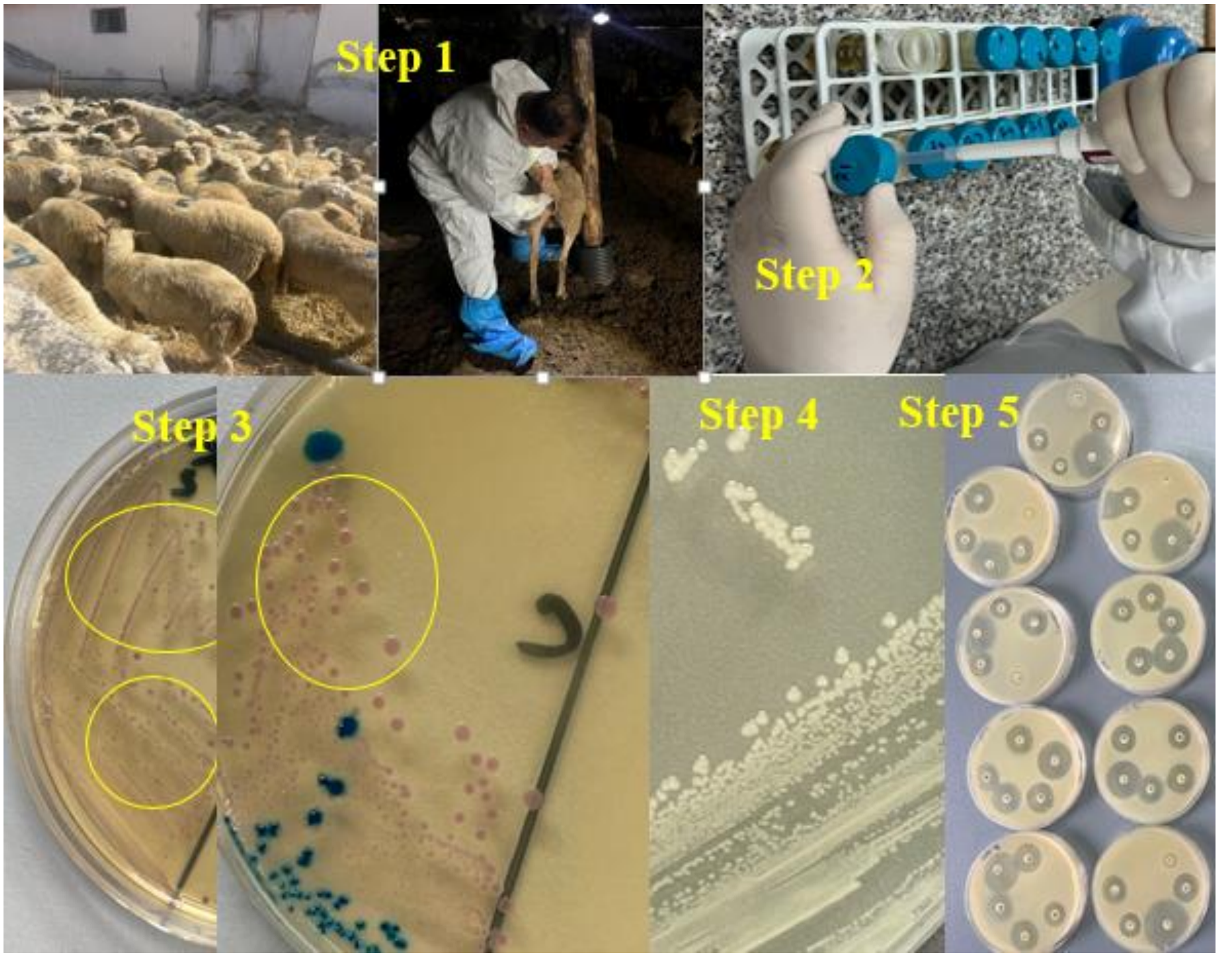

Diagram 1 – *The research approach and STEC isolation employed in this study are shown schematically. 1. Sample collection, 2. Samples inoculation and incubation* 41.5^0*C followed incubation at* 37^0*C overnight, 3. Plating inoculation* CHROMagar™ STEC*, 4. Subculture in Nutrient Agar, 5. Inoculation in liquid broth and antibiotic susceptibility test, 6. DNA extracting and culture preservation.*

Supplementary Table 1 *– Number of animals that tested E. coli STEC positive based on colony appearance on* CHROMagar™ STEC*.*

Culture results	Winter	Spring	Summer	Autumn
Weak Positive	4	6	3	7
Moderate Positive	6	7	8	8
Strong Positive	10	11	14	6
Total Positive	20	24	25	21
Total Tested	35	35	35	35
**Percentage Positive**	**57.1**	**68.6**	**71.4**	**60.0**
**(confidence intervals)**	**(40.9 to 72.0)**	**(52 to 81.4)**	**(54.9 to 83.7)**	**(43.6 to 74.4)**

Supplementary Table 2 – *Antibiotic susceptibility test results*

Samples	Amicacin	Amocycillin	Cefazolin	Cefixime	Cefoxitin	Ciprofloxacin	Doxycycline	Enrofloxacin	Nadilixic Acid	Tetracycline	Resistant
1	19.00	10.2	19.2	23.5	22.3	21.6	31.5	21.9	21.3	19.1	1
2	7.2	0	15.7	12.5	19.3	11.6	22.2	0	13.6	16.3	7
3	19.3	11.4	16.6	21.5	23.7	19.1	26.3	17.4	22.6	21.1	1
4	20.5	21.7	12.4	16.5	24.4	20.2	26.6	14.6	16.4	23.7	2
5	14.4	16.8	15.2	17.3	20.1	15.7	22.1	0	7.3	21.2	*3*
6	17.2	15.5	21.3	4.3	22.6	15.7	26.2	16.2	23.1	7.8	2
7	15.3	14.3	15.5	16.5	4.1	16.2	19.2	16.8	0	8.3	*3*
8	4.5	14.1	4.4	5.2	5.2	16.5	6.5	9.3	17.4	5.4	7
9	12.8	11.7	15.4	17.3	23.3	15.2	7.6	5.1	7.2	12.8	6
10	16.1	0	18.5	8.4	19.7	15.8	22.7	16.8	0.3	17.4	*3*
11	15.2	14.6	20.6	17.1	25.9	15.5	23.4	16.4	18.1	0	2
12	18.3	20.4	14.6	16.4	2.4	7.8	7.6	19.6	16.5	21.2	*3*
13	15.7	13.6	14.8	16.7	19.1	15.7	9.8	16.5	5.7	19.6	2
14	15.5	13.5	17.5	11.3	23.4	15.8	0	16.7	17.2	15.3	2
15	21.5	7.2	18.5	16.4	6.3	15.3	22.7	17.6	8.7	15.7	*3*
16	16.2	13.4	14.7	0	15.5	15.4	22.7	16.3	16.6	5.4	*3*
Susceptible	5	2	6	2	7	3	4	2	3	5	
Resistant	11	14	10	14	9	13	12	14	13	11	

Supplementary Table 3 – *Antibiotics breakpoints values*

Antibiotic	Susceptible (S) ≥	Resistant (R) ≤
Amikacin	18 mm	15 mm
Amoxicillin	19 mm	13 mm
Cefazolin	17 mm	14 mm
Cefixime	19 mm	16 mm
Cefoxitin	22 mm	19 mm
Ciprofloxacin	18 mm	15 mm
Doxycycline	25 mm	22 mm
Enrofloxacin	19 mm	16 mm
Nalidixic Acid	21 mm	16 mm
Tetracycline	19 mm	15 mm

Supplementary Table 4 – *Fully resistant to one, two, three, or more than three antibiotics*.

Fully Resistant to number of antibiotics	Samples	Number	Percentage
One antibiotic	1 and 3	2	12.5
Two antibiotics	4, 6, 11,13, 14	5	31.3
Three antibiotics	5, 7, 10, 12, 15, 16	6	37.5
More than three antibiotics	2, 8 and 9	3	18.8

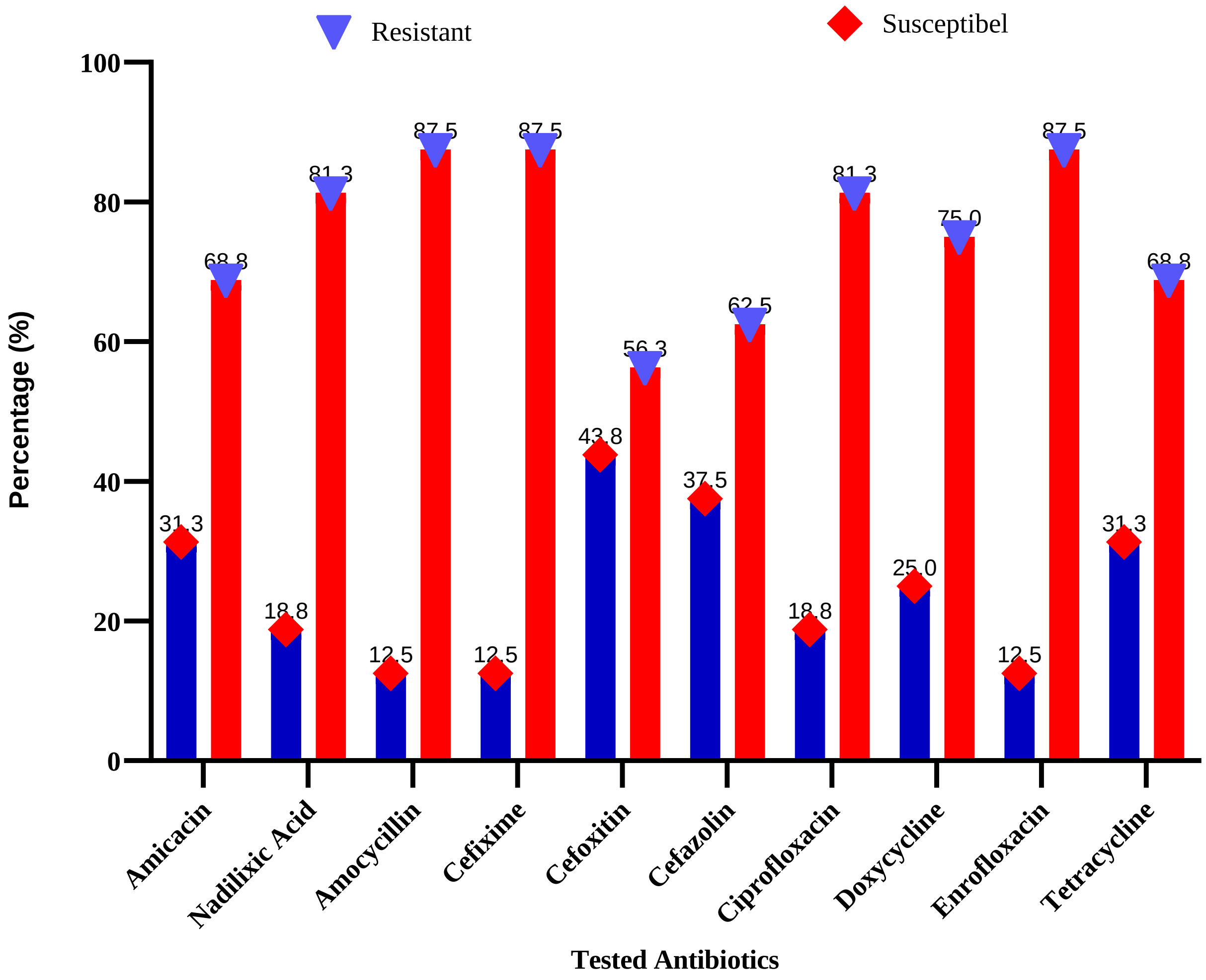

Supplementary Figure 1 – *Antibiotic susceptibility test results.*

Supplementary Table 5 – Sheep farms' external, internal, and total biosecurity scores using the Biocheck.UGent assessment tool.

	Description	Word average score	Average scores of the selected 7 sheep farms
	**External**	**External**	
A	Purchase and reproduction	61	17
B	Transport and removal of deadstock	71	38
C	Feed and water	67	39.5
D	Visitors and farmworkers	50	8.5
E	Infrastructure, location and housing	49	10
	**Internal**	**59**	**21.5**

F	Disease management	47	18.5
G	Lambing/Kidding management	47	28
H	Lamb/Kid management	67	52
I	Dairy management	65	53
J	Adult sheep/goat management	55	27.5
K	Working organisation and equipment	41	26.5
	**Subtotal internal biosecurity**	**53**	**33.5**
	**Total**	**56**	**28**

Supplementary Table 6 – External, Internal, and Total Biosecurity Scores for Seven Sheep Farms Based on the Biocheck.UGent Assessment Tool.

	**Descriptions**	**Word average**	**Farm 1**	**Farm 2**	**Farm 3**	**Farm 4**	**Farm 5**	**Farm 6**	**Farm 7**	**Average**
	**External Biosecurity**									
A	Purchase and reproduction	61	15	19	17	18	17	18	15	17
B	Transport and removal of deadstock	71	40	36	38	40	36	38	38	38
C	Feed and water	67	50	29	41	38	42	40	40	39.5
D	Visitors and farmworkers	50	7	10	8	9	8	10	8	8.5
E	Infrastructure, location and housing	49	5	15	10	12	10	10	8	10
	**Subtotal external biosecurity**	**59**	**22**	**21**	**21**	**22**	**22**	**22**	**21**	**21.4**
	**Internal**									
F	Disease management	47	18	21	18	17	22	18	16	18.5
G	Lambing/Kidding management	47	31	25	28	30	28	29	25	28
H	Lamb/Kid management	67	48	56	52	48	54	48	58	52
I	Dairy management	65	53	56	54	48	54	56	50	53
J	Adult sheep/goat management	55	38	16	28	24	28	28	30	27.5
K	Working organization and equipment	41	5	48	27	32	28	23	22	26.5
	**Subtotal internal biosecurity**	**53**	**29**	**38**	**33**	**33**	**35**	**33**	**33**	**33.5**
	**Total**	**56**	**26**	**30**	**28**	**28**	**29**	**28**	**27**	**28**
